# De-Escalation of Axillary Surgery: A Review of Choosing Wisely Guideline Evidence

**DOI:** 10.14740/wjon2710

**Published:** 2026-05-08

**Authors:** Abigail Grapes, Jessica Young, Kazuaki Takabe

**Affiliations:** a Breast Surgery, Department of Surgical Oncology, Roswell Park Comprehensive Cancer Center, Buffalo, NY 14203, USA; b Department of Surgery, University at Buffalo Jacobs School of Medicine and Biomedical Sciences, The State University of New York, Buffalo, NY 14203, USA; c Department of Immunology, Roswell Park Comprehensive Cancer Center, Buffalo, NY, USA; d Department of Breast Surgery and Oncology, Tokyo Medical University, Tokyo 160-8402, Japan; e Department of Gastroenterological Surgery, Yokohama City University Graduate School of Medicine, Yokohama 236-0004, Japan; f Division of Digestive and General Surgery, Niigata University Graduate School of Medical and Dental Sciences, Niigata 951-8520, Japan; g Department of Breast Surgery, Fukushima Medical University School of Medicine, Fukushima 960-1295, Japan

**Keywords:** Breast cancer, Lymph nodes, De-escalation, Omission, Sentinel lymph node biopsy, Choosing Wisely

## Abstract

Management of the axilla in early stage breast cancer has shifted toward less invasive approaches as evidence demonstrates equivalent oncologic outcomes with reduced morbidity. In older women with biologically favorable disease, the value of axillary staging has been increasingly questioned. This review outlines the evolution of axillary surgery from axillary lymph node dissection to sentinel lymph node biopsy and subsequent omission strategies. We synthesize the available evidence presented defining when omission of sentinel lymph node biopsy is safe and how it can be implemented in practice. The Society of Surgical Oncology 2016 Choosing Wisely^®^ guideline advises against routine sentinel lymph node biopsy in women ≥ 70 years with clinically node-negative, hormone receptor-positive/human epidermal growth factor receptor 2 (HER2)-negative invasive breast cancer receiving endocrine therapy. Across studies, omission of sentinel lymph node biopsy in appropriately selected patients is associated with low axillary recurrence rates without compromise in disease-free, breast cancer-specific, or overall survival. Although regional recurrence is modestly increased without axillary staging, these events are uncommon and typically amenable to salvage therapy. Despite strong evidence and professional endorsement, implementation of this recommendation remains inconsistent, with persistently high utilization of sentinel lymph node biopsy among women eligible for omission. Contemporary data indicate that adjuvant therapy decisions in hormone receptor-positive disease are driven primarily by tumor biology, with nodal status altering management in a minority of postmenopausal women. We propose a structured clinical decision framework incorporating tumor biology, physiologic rather than chronological age, competing mortality risk, and planned endocrine therapy. When nodal status is unlikely to influence treatment, omission of sentinel lymph node biopsy is a safe, evidence-based refinement that reduces morbidity without compromising survival. Multidisciplinary coordination is essential to optimize adjuvant therapy while minimizing low-value surgical care.

## Introduction

Advances in breast cancer screening and systemic therapy have shifted the management of early stage breast cancer toward increasingly individualized and less invasive treatment strategies. Earlier detection has enabled excellent locoregional control with smaller surgical interventions, prompting re-evaluation of procedures that provide limited therapeutic benefit while contributing to morbidity, particularly in older patients with favorable tumor biology. Axillary surgery has been a central focus of this de-escalation paradigm.

Historically, axillary lymph node dissection (ALND) served both diagnostic and therapeutic roles. However, multiple randomized trials demonstrated no survival advantage to extensive axillary surgery in clinically node-negative patients, establishing that nodal surgery primarily provides prognostic information rather than therapeutic benefit in this setting [1–6]. These findings led to the widespread adoption of sentinel lymph node biopsy (SLNB) as a less morbid staging alternative. Subsequent studies further questioned the necessity of SLNB in select low-risk populations.

Elderly women with early stage, hormone receptor (HR)-positive/human epidermal growth factor receptor 2 (HER2)-negative breast cancer represent a population in whom the risks of axillary staging may outweigh its benefits. These patients experience excellent breast cancer-specific outcomes, face substantial competing mortality from comorbid conditions, and derive little survival benefit from knowledge of axillary nodal status [7–10]. This ultimately laid the foundation for the Choosing Wisely^®^ guideline which discouraged routine SLNB in women ≥ 70 years old with clinically node-negative (cN0), early stage, HR-positive/HER2-negative invasive breast cancer [11]. This review examines the evolution of axillary surgery with a specific focus on the evidence supporting omission of SLNB in women over 70 years of age with early stage, HR-positive/HER2-negative breast cancers. We synthesize data on oncologic outcomes, recurrence, morbidity, patient selection, interpretation of undertreatment signals, the relationship between SLNB omission and adjuvant therapy use, and real-world adoption of the Choosing Wisely^®^ guideline. We further propose a structured clinical decision framework integrating tumor biology, physiologic age, competing mortality risk, and endocrine therapy planning to guide safe implementation of SLNB omission.

## Historical Context: De-Escalation as a Foundation for Omission of Axillary Surgery

The Halsted radical mastectomy, involving *en bloc* removal of the breast, pectoralis muscles, and axillary contents, was historically the standard surgical procedure for breast cancer regardless of stage [12]. Although effective for local control, this approach was associated with substantial morbidity without clear survival benefit compared with less extensive surgery, promoting investigation into de-escalation strategies [13]. The landmark National Surgical Adjuvant Breast and Bowel Project (NSABP) B-04 trial challenged the therapeutic necessity of aggressive axillary surgery by randomizing clinically node-negative patients to radical mastectomy, total mastectomy with regional radiation, or total mastectomy alone [14]. After 25-year follow-up, no significant differences were observed in disease-free survival (DFS), recurrence-free survival (RFS), distant disease-free survival (DDFS), or overall survival (OS), demonstrating no survival advantage from removal of occult nodal disease or regional nodal irradiation [15]. These findings established that axillary surgery primarily provides staging rather than therapeutic benefit in clinically node-negative patients.

Subsequent trials extended surgical de-escalation to the breast. NSABP B-06 demonstrated that lumpectomy with radiation achieved survival equivalent to mastectomy in women with stage I–II disease, establishing breast conserving surgery (BCS) as standard treatment [16]. Although all patients underwent ALND, this trial reinforced that increasingly conservative surgical approaches could maintain oncologic safety.

The INT09/98 trial further questioned the necessity of axillary surgery by randomizing patients with T1N0 breast cancer to breast conserving surgery with or without ALND [17]. After 10 years, there were no differences in DFS or OS, suggesting that systemic therapy decisions could be guided by tumor biology rather than nodal staging.

Despite this, axillary staging remained standard practice because nodal status provides important prognostic information and historically influenced adjuvant therapy recommendations [18–20]. However, ALND is associated with substantial morbidity, motivating development of less invasive staging techniques.

SLNB, first developed in melanoma and applied to breast cancer in the 1990s, enabled accurate staging with reduced morbidity by selectively removing the first draining lymph node [21–25]. Multiple randomized trials subsequently confirmed that SLNB could safely replace ALND in clinically node-negative patients without compromising survival [26]. Several landmark randomized controlled trials that provided the foundation for de-escalation are summarized here (Supplementary Material 1, wjon.elmerpub.com).

Further de-escalation was supported by trials demonstrating that completion ALND could be safely omitted even in selected patients with nodal metastases. In NSABP B-32, which randomized 5,611 clinically node-negative patients undergoing lumpectomy or mastectomy to SLNB plus ALND versus SLNB alone, SLNB alone provided equivalent OS, DFS, and regional control compared with ALND in node-negative patients [5]. Similarly, the International Breast Cancer Study Group (IBCSG) 23-01 and AATRM 048 trials demonstrated no survival benefit to completion ALND in patients with micrometastatic nodal disease [1, 2, 6].

In patients with limited macrometastatic nodal involvement, ACOSOG Z0011 established that completion ALND could be safely omitted in women with cT1–2N0 breast cancer and 1–2 positive sentinel nodes undergoing BCS with radiation and systemic therapy, without compromising survival or regional control [3, 4]. While practice-changing in nature, critics of this trial raised concerns that incidental axillary radiation likely contributed to regional control. The findings from Z0011 were confirmed by subsequent trials, including SINODAR-ONE and SENOMAC, which expanded eligibility to include mastectomy patients and demonstrated continued oncologic safety with omission of ALND [27, 28]. The SERC trial then further expanded inclusion criteria to cT0–2N0 breast cancer with any number of positive SLNs, survival results remain pending [29].

Finally, trials such as After Mapping of the Axilla: Radiotherapy or Surgery (AMAROS) and Optimal Treatment of the Axilla- Surgery Or Radiotherapy (OTOASOR) demonstrated that axillary radiotherapy provides equivalent regional control compared with ALND while reducing morbidity [30, 31]. Notably, the AMAROS trial demonstrated comparable regional control and survival at 10-year follow-up, confirming the long-term safety of axillary treatment de-escalation.

**Figure 1:**
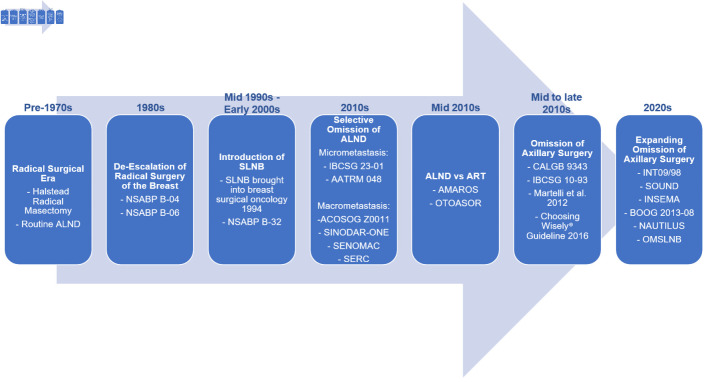
Evolution of axillary management in breast cancer. Axillary surgery has progressively de-escalated from routine axillary lymph node dissection (ALND) toward sentinel lymph node biopsy (SLNB), and more recently, omission of axillary surgery in selected patients. Landmark trials, including NSABP B-04 [15], NSABP B-06 [16], NSABP B-32 [5], IBCSG 23-01 [1], AATRM 048 [6], ACOSOG Z0011 [4], SINODAR-ONE [27], SENOMAC [28], SERC [29], AMAROS [31], OTOASOR [30], CALGB 9343 [8], IBCSG 10-93 [9], Martelli et al (2012) [32], INT09/98 [17], SOUND [33], and INSEMA [34], have established the safety of reducing surgical intervention without compromising oncologic outcomes. BOOG 2013-08 [35], NAUTILUS [36], and OMSLNB [37] trials are ongoing to confirm preliminary findings of SOUND and INSEMA expanding the population of women eligible for omission of axillary surgery. NSABP: National Surgical Adjuvant Breast and Bowel Project; IBCSG: International Breast Cancer Study Group; AMAROS: After Mapping of the Axilla: Radiotherapy or Surgery; OTOASOR: Optimal Treatment of the Axilla- Surgery Or Radiotherapy; SOUND: Sentinel Node vs. Observation After Axillary Ultra-Sound; INSEMA: Intergroup Sentinel Mamma; CALGB: Cancer and Leukemia Group B.

Taken together, these landmark trials established a progressive paradigm shift in axillary management: from routine radical dissection, to selective staging with SLNB, to omission of further axillary treatment in selected patients. Figure 1 highlights this historical progression [1, 4–6, 8, 9, 15–17, 27–37]. This evolution demonstrated that axillary surgery largely serves a staging function, with limited therapeutic value in biologically favorable disease. These findings laid the foundation for contemporary SLNB omission strategies. While oncologic equivalence supported de-escalation, the morbidity of axillary surgery further strengthened the rationale for limiting its use.

## Morbidity of Axillary Surgery as a Rationale for Omission

Axillary surgery is associated with well-established morbidity, including lymphedema, chronic pain, sensory deficits, shoulder dysfunction, and diminished quality of life. These complications may be particularly consequential in older adults with baseline frailty or comorbid conditions, in whom functional independence and quality of life may be more important than small differences in disease recurrence. The recognition that axillary surgery provides limited therapeutic benefit in biologically favorable disease has intensified efforts to minimize these harms.

ALND carries a higher risk of complications compared to SLNB, with morbidity further exacerbated by the addition of axillary radiation [38]. Lymphedema occurs in approximately 15–25% of patients following ALND, compared with substantially lower rates after SLNB [38, 39]. Strong risk factors for development of lymphedema include ALND, mastectomy, higher number of lymph nodes removed, and elevated body mass index (BMI). Meanwhile, moderate evidence implicates chemotherapy, radiotherapy, nodal metastases, and physical inactivity [39]. Although SLNB significantly reduces morbidity compared with ALND, it is not without risk. Lymphedema rates following SLNB range from approximately 3–8%, and patients may still experience pain, sensory changes, and reduced arm function [38, 39].

Patient-reported outcome studies further highlight the impact of axillary surgery. In the Axillary Lymphatic Mapping Against Nodal Axillary Clearance (ALMANAC) trial comparing quality of life outcomes between SLNB and ALND in early stage, clinically node-negative patients, SLNB was associated with significantly shorter operative time, reduced drain use and hospital stay, faster recovery of normal activities, lower rates of arm swelling and sensory deficits, and improved overall quality of life [40]. These findings underscore a critical principle in modern axillary management: when nodal staging does not influence treatment decisions, even the relatively low morbidity of SLNB represents avoidable harm.

The morbidity associated with axillary surgery, combined with its limited therapeutic value in selected patients, provides a strong rationale for omission of SLNB when oncologically safe. The convergence of limited therapeutic benefit and measurable morbidity provided the basis for formal guideline recommendations discouraging routine SLNB in appropriately selected women.

## Omission of Axillary Surgery in Postmenopausal Women

### Choosing Wisely^®^

In 2012, the American Board of Internal Medicine (ABIM) launched the Choosing Wisely^®^ campaign to encourage critical conversations between clinicians and patients regarding tests and procedures that may offer limited clinical value. In 2016, the Society of Surgical Oncology (SSO) issued its Choosing Wisely^®^ recommendation aimed at reducing low-value breast care, advising against routine SLNB in women ≥ 70 years of age, who are clinically node-negative with early stage, HR-positive, HER2-negative invasive breast cancer [11]. Their recommendation was informed by evidence from four key studies: CALGB 9343, IBCSG trial 10-93, Martelli et al (2011) [10], and Chung et al (2015) [7], which collectively demonstrated that, in women receiving endocrine therapy, omission of SLNB does not worsen breast cancer-specific mortality or increase locoregional recurrence [7–10]. SLNB remains appropriate when nodal status would alter adjuvant therapy.

The American Society of Breast Surgeons similarly supports omission of axillary staging in women ≥ 70 years old with cT1–2N0, HR-positive breast cancer, and when nodal findings would not alter adjuvant therapy due to advanced age or comorbidities [41]. Consistent with this, the National Comprehensive Cancer Network (NCCN) guidelines endorse the SSO choosing Wisely^®^ recommendation for patients ≥ 70 years old with pT1, cN0, HR-positive/HER2-negative invasive breast cancer and has recently expanded this to appropriately selected postmenopausal patients > 50 years old with HR-positive/HER2-negative, cT1N0 disease verified by axillary ultrasound, grade 1–2, agreeable to receiving whole breast radiation therapy (RT) and endocrine therapy based on data from the Sentinel Node vs. Observation After Axillary Ultra-Sound (SOUND) and Intergroup Sentinel Mamma (INSEMA) trials [42]. Table 1 summarizes current professional society recommendations supporting omission of SLNB [11, 41, 42].

**Table 1:** Current Professional Society Guidelines Supporting Omission of SLNB

SSO Choosing Wisely^®^ [11]	Avoid routine SLNB in women ≥ 70 years of age with cN0, early stage, HR+, HER2– invasive breast cancer treated with endocrine therapy.
American Society of Breast Surgeons [41]	Axillary lymph node staging is not indicated in women ≥ 70 years of age with cT1–2N0 HR+ breast cancer and when it will not affect adjuvant treatment recommendations.
National Comprehensive Cancer Network [42]	Axillary staging may be omitted in patients with favorable tumor biology, significant comorbidities, or when adjuvant therapy decisions will not be impacted. Based on SOUND and INSEMA trials, patients > 50 years and postmenopausal with cT1N0 (by axillary US), with HR+/HER2–, grade 1–2 tumors agreeable to receiving whole breast RT and endocrine therapy.

SSO: the Society of Surgical Oncology; SLNB: sentinel lymph node biopsy; HR: hormone receptor; HER2: human epidermal growth factor receptor 2; SOUND: Sentinel Node vs. Observation After Axillary Ultra-Sound; INSEMA: Intergroup Sentinel Mamma; US: ultrasound.

### Impact of omission of axillary surgery in postmenopausal women on recurrence and survival outcomes

The Choosing Wisely^®^ recommendation to omit routine SLNB in women ≥ 70 years of age with cN0, early stage, HR-positive/HER2-negative breast cancer reflects a broader shift toward evidence-based de-escalation of axillary surgery. This approach is supported by a substantial body of clinical research demonstrating that, in carefully selected older patients, omission of axillary surgery minimally increases axillary and regional recurrence while not adversely affecting disease-free, breast cancer-specific, or overall survival, while reducing surgical morbidity. The following is a review of the pivotal randomized trials and observational cohorts that have evaluated omission of axillary surgery in this population, focusing on oncologic outcomes and recurrence data. A structured summary of the key randomized and observational studies evaluating oncologic outcomes after omission of axillary surgery in postmenopausal women is provided here (Supplementary Material 2, wjon.elmerpub.com). For clarity, recurrence terminology is used as follows: “local recurrence” denotes relapse confined to the ipsilateral breast, “axillary recurrence” refers to nodal relapse confined to the ipsilateral axilla, “regional recurrence” includes the addition of supraclavicular or infraclavicular nodal events, “locoregional recurrence” encompasses breast/chest wall, axillary, supraclavicular, and infraclavicular nodal recurrence, and “distant metastasis” is disease outside of the ipsilateral breast, contralateral breast, and regional lymph nodes.

One of the earliest studies informing this approach was Cancer and Leukemia Group B (CALGB) 9343, a randomized trial evaluating adjuvant radiation after lumpectomy and tamoxifen in women ≥ 70 years old with cT1N0, estrogen receptor (ER)-positive breast cancer [43]. Axillary surgery was not mandated, and more than 60% of patients did not undergo nodal staging; however, regional recurrence rates remained low. At 10 years, radiation improved locoregional control (98% vs. 91%, P < 0.001 by Kaplan–Meier and log-rank testing), but there were no significant differences in distant metastasis, breast cancer-specific survival (BCSS), or OS [8]. Regional recurrences were uncommon in patients who did not undergo axillary surgery and were successfully managed with delayed treatment. Only 6% of the study population died from breast cancer, indicating that non-cancer causes accounted for most deaths in this elderly population. These findings suggest that omission of axillary surgery may be safe in selected older women with early stage, ER-positive breast cancer receiving endocrine therapy, particularly when nodal status would not alter systemic treatment. This study contributed to subsequent investigations evaluating de-escalation of axillary management in low-risk patients.

Subsequent randomized trials directly compared axillary surgery with omission in postmenopausal women. Martelli et al randomized women ages 60–85 years with cT1N0 breast cancer to lumpectomy with or without ALND; all received adjuvant radiation and tamoxifen [32, 44]. At 5-year follow-up, there were no significant differences in OS, breast cancer mortality, or crude cumulative incidence of breast events. Axillary recurrence occurred in only 1.8% of patients who did not undergo ALND [44]. At 15-year follow-up, the crude cumulative incidence of axillary recurrence rose modestly to 6% in the no ALND group—two cases were successfully managed with delayed ALND, while two patients developed distant metastasis and died of disease. Small differences in ipsilateral breast tumor recurrence, axillary recurrence, and distant metastasis between the two groups were not statistically significant and did not translate into survival differences. Importantly, ER-negative tumors were associated with a markedly higher risk of distant metastasis and breast cancer-related death—approximately three- to fivefold higher, respectively—compared to ER-positive disease, highlighting a population less amenable to omission of axillary surgery [32]. Overall, the study demonstrated that elderly women treated with lumpectomy, adjuvant radiation, and tamoxifen derived no survival benefit from ALND and, given the low incidence of axillary recurrence, this finding supports omission of axillary surgery in this population.

The IBCSG trial 10-93 was a prospective, multicenter, randomized controlled trial evaluating OS and DFS after omission of axillary surgery [9]. Due to limited accrual, the study was underpowered for survival endpoints and focused on quality-of-life measures. From 1993 to 2002, 473 women ≥ 60 years old with cT1–3N0M0 breast cancer, regardless of ER status, were randomized to breast surgery (mastectomy or BCS) with or without axillary clearance, followed by 5 years of tamoxifen. Quality of life was evaluated using patient self-assessments preoperatively and up to 6 years postoperatively, along with physician assessments of ipsilateral arm mobility, pain, daily function, and arm circumference up to 2 years. Most patients (80%) had ER-positive disease, and 56% had T1 tumors. Axillary clearance was associated with worse early quality of life, including restricted arm movement and pain at the first postoperative assessment (39% vs. 15%, P = 0.000001, calculated by analysis of variance), but differences resolved over time. After 6 years, there were no significant differences in DFS or OS, consistent across ER subgroups. The trial concluded that omitting axillary surgery does not compromise survival in clinically node-negative, postmenopausal women, and despite transient quality of life benefits, the findings support avoiding axillary surgery, particularly in ER-positive patients receiving adjuvant endocrine therapy [9].

A meta-analysis by Liang et al [45] of randomized controlled trials published through 2014 compared outcomes of axillary surgery (SLNB or ALND) versus omission in women ≥ 70 years old with cT1–2N0 invasive breast cancer. Only two eligible trials met inclusion criteria, both of which were previously discussed [9, 32]. Among 692 patients, ALND was associated with a reduced risk of axillary recurrence compared to omission (relative risk (RR), 0.24; 95% confidence interval (CI), 0.06–0.95; I^2^ = 0%; P = 0.04 by fixed-effect meta-analysis), but there were no significant differences in local recurrence, distant recurrence, DFS, or OS [45]. These results suggest that although omission of axillary surgery may increase axillary recurrence, it does not adversely affect survival, supporting de-escalation in this patient population.

Several retrospective studies have compared axillary surgery versus omission outcomes in postmenopausal women. In a retrospective study with 15-year follow-up, Martelli et al evaluated 671 women ≥ 70 years old with clinically node-negative breast cancer (pT1–pT4b), predominantly HR-positive, treated with lumpectomy with or without ALND [10]. Approximately one-third received adjuvant breast radiation, and all received tamoxifen for at least 2 years. Among those undergoing ALND, 33.7% had pathologically positive nodes, including 29% with pT1 tumors. In the no-ALND group, the 15-year cumulative incidence of axillary recurrence was 5.8% overall and 3.7% among pT1 tumors, compared to none in the ALND group. Breast cancer-specific mortality did not differ between groups. Higher distant metastasis rates were observed among patients with lobular histology, tumor size > pT1, and ER-negative disease, suggesting that omission of axillary staging may be inappropriate in these subgroups [10].

Chung et al reviewed a prospectively maintained database of 140 women ≥ 70 years old with cT1–2N0 breast cancer managed with BCS without SLNB between 2000 and 2011 [7]. This cohort primarily had small, HR-positive ductal tumors. During follow-up, there was one axillary recurrence in a patient with triple-negative breast cancer and four breast cancer-related deaths, all associated with unfavorable tumor biology. Five-year OS was 70%, and BCSS was 96% [7]. These findings suggest omission of SLNB does not compromise survival in elderly patients, whose mortality is more often related to comorbidities.

Poodt et al analyzed Netherlands Cancer Registry data comparing incomplete axillary staging (no axillary staging or SLNB without cALND if positive) versus complete axillary staging (negative SLNB or cALND when positive) in women ≥ 75 years old with invasive breast cancer treated with BCS or mastectomy from 2001 to 2008 [46]. Incomplete staging was more common in older patients with greater comorbidity. At 10 years, regional recurrence rate was 5.2% with incomplete staging, with no difference in OS (28.5% vs. 32.7%), despite low absolute survival due to advanced age (mean 81.7 vs. 80.1). In addition, breast cancer accounted for 10.8% of deaths at 10 years, compared to 64.5% from non-cancer causes in the incomplete axillary staging group supporting omission of axillary surgery in older, comorbid patients [46].

Cha et al analyzed Korean Breast Cancer Registry data from 2005 to 2014, comparing women ≥ 70 years old with clinically node-negative invasive breast cancer who underwent BCS or mastectomy with or without axillary surgery (SLNB, SLNB + ALND, or ALND alone) [47]. After matching, there were no significant differences in 5-year OS (86.9% vs. 85.2%) or BCSS (94.7% vs. 96.7%) among patients undergoing or omitting axillary surgery, respectively. Axillary staging did not improve survival regardless of chemotherapy, radiotherapy, or endocrine therapy use [47].

Corso et al performed a matched cohort study of women ≥ 70 years old with primary invasive breast cancer treated at the European Institute of Oncology from 1994 to 2008 [48]. Axillary surgery was omitted in selected patients who were > 80 years old, with cN0, specific comorbidities, early stage tumors, and favorable biopsy features. Patients undergoing axillary surgery (SLNB and/or ALND) were matched 1:1 with those who did not. There were no significant differences in ipsilateral breast tumor recurrence, distant metastasis, or contralateral breast cancer. However, axillary recurrence was significantly higher in the no-axillary surgery group at 5 and 10 years (P = 0.038) calculated using the Gray’s test, but without impact on survival. Increased regional recurrence was associated with poorly differentiated, highly proliferative (Ki67 ≥ 20%), and luminal B tumors. The authors concluded that axillary surgery may be safely omitted in selected elderly women with low risk, T1, ER-positive breast cancers [48].

While most prior studies predominantly included HR-positive patients, many also enrolled women with HER2-positive and triple negative disease. To better evaluate the feasibility of omitting axillary surgery in a lower-risk subgroup, several studies have specifically focused on postmenopausal women with ER-positive/HER2-negative invasive breast cancer.

O’Connell et al evaluated postmenopausal women with low-risk invasive breast cancers treated from 1995 to 2006 with mastectomy or BCS plus adjuvant radiation, without axillary staging [49]. Low-risk features included ER-positive tumors < 20 mm and grade 1, or < 15 mm and grade 2, without lymphovascular invasion. All patients received 5 years of adjuvant endocrine therapy. Axillary recurrence rates were low (0.8% at 5 years and 1.9% at 10 years), with favorable survival outcomes: DFS of 96.6% and 91.2%, distant DFS of 99.2% and 97%, and OS of 90.3% and 75.5% at 5 and 10 years, respectively [49].

Carleton et al retrospectively compared women ≥ 70 with early stage ER-positive, HER2-negative, clinically node-negative breast cancer treated with or without SLNB [50]. At a median follow-up of 4.1 years, locoregional recurrence occurred in 3.5% of the SLNB group and 4.5% in the no-SLNB group, with no difference in locoregional recurrence-free survival (LRFS). Although DFS was numerically higher without SLNB, the difference was not significant. Among patients undergoing SLNB, 11.5% were node-positive, but nodal status was not associated with DFS or LRFS, suggesting limited clinical benefit of SLNB in this population [50].

Chung et al prospectively evaluated women ≥ 65 years old with cT1–2 N0 (by preoperative axillary ultrasound), ER-positive/HER2-negative breast cancer treated with BCS without SLNB followed by endocrine therapy [51]. Axillary recurrence occurred in 1.6% of patients. At 3-year follow-up, RFS was 98.2%, DFS was 91.2%, BCSS was 91.2%, and OS was 94.8%. Nonadherence to endocrine therapy was the only factor significantly associated with recurrence, highlighting the importance of endocrine adherence in this population [51].

Egbert et al retrospectively compared women 70 years old with pT1, cN0, ER-positive/HER2-negative breast cancer treated with or without SLNB and found no differences in local, axillary, distant, locoregional recurrence, or DFS at a median follow-up of 2.9 years [52]. Similarly, in a meta-analysis by Lai et al including eight studies of women ≥ 65 years old with cT1–2N0, ER-positive/HER2-negative breast cancer undergoing BCS demonstrated no differences in OS or BCSS between SLNB and omission, without increased recurrence risk [53]. Collectively, these studies support the oncologic safety of omitting SLNB in selected postmenopausal women with low risk, cT1–2N0, ER-positive/HER-negative breast cancer, showing no significant survival disadvantage and emphasizing the critical role of endocrine therapy adherence in reducing recurrence risk.

Building on this, Korzets et al evaluated women ≥ 70 years old with cT1N0, HR-positive/HER2-negative breast cancer treated with lumpectomy omitting both SLNB and adjuvant RT, while receiving adjuvant endocrine therapy [54]. In this small cohort (n = 100), 7% developed recurrence (four local, two locoregional, and one distant) at a median follow-up of 3.9 years. Median DFS was 42 months, and no breast cancer-related deaths occurred. Improved DFS was observed in tumors < 13 mm [54]. These findings suggest that omission of both SLNB and radiation may be feasible in carefully selected patients, though validation in larger cohorts is warranted. Most ongoing SLNB omission clinical trials continue to include adjuvant radiation as part of the treatment protocol.

Despite generally favorable results, some studies suggest omission of axillary staging may be associated with worse OS, potentially reflecting undertreatment or selection bias. Castelo et al evaluated women ages 65–95 years old with stage I–II breast cancer and found worse OS among those omitting axillary staging on both unadjusted and adjusted analyses, including subgroups ≥ 70 years of age, ER-positive/HER2-negative disease, lobular histology, and comorbidity score of ≥ 1. However, BCSS did not differ after adjustment, indicating that the OS difference was likely driven by non-cancer mortality [55].

Using the National Cancer Database (NCDB), Tamirisa et al compared women ≥ 70 years old with cT1–3N0 breast cancer who did or did not undergo axillary staging [56]. Nodal metastases were identified in 14% of staged patients. Axillary staging was associated with greater use of adjuvant therapy, while omission was associated with worse OS (median 111 vs. 74.5 months), including after adjustment in low-risk subgroups (cT1, grade 1–2, ER-positive disease: OS 121.5 vs. 85.7 months) [56]. Similarly, a Surveillance, Epidemiology, and End Results Program (SEER) analysis by Castelo et al found that omission of axillary staging reduced receipt of adjuvant radiation or chemotherapy and was associated with worse OS and BCSS, particularly in women < 80 years [57]. Collectively, these findings highlight a potential risk of undertreatment among older patients when axillary surgery is omitted, emphasizing the importance of multidisciplinary evaluation, careful assessment of comorbidities and functional status, and individualized treatment planning to balance oncologic safety with quality of life.

### Prevalence of nodal positivity

A key concern when omitting SLNB is the prevalence of occult nodal metastasis and its potential clinical implications. Several studies have examined nodal positivity rates in early stage ER-positive/HER2-negative breast cancer to inform SLNB omission. Downs-Canner et al, using NCDB data, reported that nodal metastases decrease with age, with < 10% of women ≥ 70 years old with cT1N0 disease demonstrating nodal involvement, independent of histologic subtype, race, or comorbidities [58]. McKevitt et al found a 25% SLNB positivity rate among women ≥ 70 years old; however, 5-year BCSS was similar between node-negative and node-positive patients receiving endocrine therapy, while omission of endocrine therapy in node-positive patients was associated with worse BCSS. Women aged 75–79 with ≤ 2 cm, grade 1–2 tumors had 5-year BCSS > 95% regardless of SLNB status or endocrine therapy adherence [59].

Matar et al reported SLNB positivity in 21% of women ≥ 70 years with HR-positive/HER2-negative breast cancer undergoing mastectomy (1% ductal carcinoma *in situ* (DCIS), 13% in cT1, and 29% in cT2), with a higher nodal burden in cT2 disease (3% rate of pN2–3 in cT1 vs. 27% in cT2) [60]. Larger tumor size, high grade, and lymphovascular invasion predicted nodal involvement, yet nodal status did not affect LRFS, supporting omission of SLNB in women ≥ 70 years with cTis or cT1N0 HR-positive/HER2-negative disease undergoing total mastectomy [60]. In contrast, Davey et al observed a low SLNB positivity rate of 2.1% in postmenopausal women (age 48–90) with HR-positive/HER2-negative, cN0 breast cancer treated with BCS or total mastectomy with SLNB. Nodal status did not influence recurrence or mortality. Instead, tumor genomic risk score was the primary determinant of chemotherapy use in this population [61].

Collectively, nodal metastasis rates are generally low in women ≥ 70 years old with cT1N0 HR-positive/HER2-negative breast cancer, identifying a subgroup in whom omission of SLNB may be appropriate. However, nodal positivity increases with tumor size and adverse pathologic features, warranting caution when nodal information may influence systemic therapy decisions. Emerging biomarkers may further refine risk stratification; for example, low RUFY3 expression levels have been associated with nodal positivity and worse disease-specific survival in older women [62]. Overall, integration of clinical, pathologic, and molecular factors may enable more individualized de-escalation of axillary surgery.

## Impact of SLNB on Adjuvant Therapy

### Rates of adjuvant therapy in patients with a positive SLNB

Adjuvant therapy decisions in early-stage HR-positive breast cancer are increasingly driven by tumor biology rather than nodal status alone. Nevertheless, pathologic axillary staging continues to influence treatment patterns in clinical practice. Multiple observational studies demonstrate that patients undergoing SLNB, particularly those with positive nodes, are significantly more likely to receive adjuvant endocrine therapy, radiation, and chemotherapy [59, 63–68].

McKevitt et al reported that a positive SLNB was independently associated with increased odds of chemotherapy (odds ratio (OR) = 6.4; 95% CI, 4.1–10.0; P < 0.001), endocrine therapy (OR = 2.3; 95% CI, 1.8–3.0; P < 0.0001), and radiation therapy (OR = 4.9; 95% CI, 3.9–6.3; P < 0.001) by multivariable Cox regression modeling. Nodal radiation and breast or chest wall radiation were also more frequently administered following lumpectomy or mastectomy [59]. Similarly, Chagpar et al reported that lymph node evaluation (SLNB or ALND) and pathologically positive nodes were associated with higher rates of chemotherapy (28.3% vs. 5.5% for negative SLNB, P < 0.001), endocrine therapy (83.6% vs. 71.4%, P < 0.001), and radiation therapy after lumpectomy (81.4% vs. 73.6%, P < 0.001) or mastectomy (30.3% vs. 5.1%, P < 0.001), although statistical methods were not specified [63].

Consistent with these findings, Laws et al observed that patients with axillary metastases were more likely than node-negative patients to receive nodal radiation following lumpectomy (53.1% vs. 0.7%, P < 0.001) or mastectomy (45.0% vs. 2.2%, P < 0.001), as well as systemic therapy, including endocrine therapy (81.9% vs. 69.1%, P = 0.002) or combined chemotherapy and endocrine therapy (6.9% vs. 2.1%, P = 0.002) [64]. Di Lena et al similarly found that positive SLNB status was associated with higher use of local radiation therapy (77.8% vs. 50.6%, P = 0.012), regional radiation therapy (40.9% vs. 3.9%, P < 0.001), and adjuvant chemotherapy (14.8% vs. 3.3%, P = 0.029) [66]. All statistical significance testing was assessed by Chi-square or Fisher’s exact test.

A meta-analysis by Daly et al, including 10 retrospective studies, confirmed that positive SLNB was associated with increased odds of receiving adjuvant chemotherapy (OR, 4.64; 95% CI, 3.18–6.77; P < 0.00001) and radiation therapy (OR, 1.71; 95% CI, 1.18–3.27; P = 0.005), calculated using the Mantel-Haenszel method [65]. Collectively, these studies indicate that pathologic axillary nodal status remains a key determinant of adjuvant therapy use, with positive SLNB strongly influencing both systemic and radiation treatment decisions.

### Impact of SLNB omission on adjuvant therapy and interpretation of undertreatment signals

Concerns have been raised that omission of SLNB may result in undertreatment. Importantly, however, lower use of adjuvant therapy among unstaged patients should not be interpreted as evidence that SLNB omission itself causes undertreatment. In a Cedars-Sinai cohort of women ≥ 70 years old with cT1–2N0 breast cancer undergoing BCS without SLNB, chemotherapy, radiation, and endocrine therapy were omitted more frequently compared with staged patients (98%, 76%, and 59%; all P < 0.05 by Chi-square) [7]. Similarly, Castelo et al in a population-based cohort study demonstrated lower rates of endocrine therapy, chemotherapy, and breast or chest wall radiation among patients omitting axillary surgery, although axillary radiation was more common. OS was lower in omission cohorts, but BCSS did not differ after adjustment, suggesting that competing mortality and comorbidity explain much of the observed survival difference [55].

Additional studies confirm lower initiation and adherence to endocrine therapy with increasing age, as well as reduced use of adjuvant radiation among women omitting axillary staging [66, 69–71]. While this pattern raises concern when multiple de-escalation strategies occur simultaneously, it does not establish a causal relationship between SLNB omission and systemic undertreatment [72]. In selected cases, absence of nodal information may also limit eligibility for partial breast irradiation protocols requiring pathologic node-negative confirmation [73].

Two explanations merit consideration. First, SLNB may theoretically influence treatment decisions by reinforcing perceived oncologic risk. However, contemporary evidence suggests that this effect is limited in older patients with HR-positive disease. Wanis et al, analyzing 119,312 patients from the NCDB with cT1N0 HR-positive breast cancer undergoing upfront BCS, found that SLNB results altered systemic therapy recommendations in fewer than 10% of postmenopausal patients [74]. Similarly, the SOUND trial demonstrated no significant differences in adjuvant therapy use based on availability of SLNB information [33]. Institutional studies from Mayo Clinic and Dana-Farber Brigham Cancer Center have reported similar findings [71, 75]. In the absence of SLNB, adjuvant treatment decisions increasingly rely more heavily on tumor biology and clinicopathologic risk factors. In the INT09/98 trial, chemotherapy recommendations were guided by biologic risk stratification incorporating receptor status and high-risk features, including ER-negative disease, or ER-positive tumors with adverse histopathologic characteristics such as grade III histology, HER2-positivity, or lamin receptor positivity [17]. Similarly, van Roozendaal et al demonstrated that inclusion of pathologic nodal status altered systemic therapy recommendations in only 1% of cases when modeled using Adjuvant! Online, which incorporated patient age, comorbidity, tumor characteristics, and nodal information [76]. Together, these data emphasize the shift in adjuvant therapy decision-making in HR-positive disease toward tumor biology and clinicopathologic risk factors, with nodal staging influence management in a minority of older patients.

Second, SLNB may function as a surrogate marker for frailty rather than a cause of undertreatment. In multiple population-based studies, SLNB omission is strongly associated with advanced age, higher comorbidity burden, and limited life expectancy [68, 77, 78]. When applied within evidence-based criteria, specifically in node-negative, postmenopausal patients with HR-positive breast cancers, survival outcomes are not compromised [9, 10, 32, 44, 46–49, 51, 52]. Lower adjuvant therapy use in omission cohorts therefore likely reflects individualized treatment decisions based on patient age, comorbidity burden, and competing mortality risk rather than absence of nodal staging itself.

### Endocrine therapy as the central determinant of safety

Endocrine therapy remains the cornerstone of adjuvant systemic treatment for hormone-receptor positive breast cancer. Tamoxifen substantially reduces recurrence and breast cancer mortality, regardless of nodal status, and aromatase inhibitors provide additional benefit in postmenopausal women [79–81]. Conversely, non-adherence is associated with increased recurrence risk, including in patients who omit SLNB [51].

Accordingly, the safety of SLNB omission depends less on nodal staging and more on initiation and adherence to endocrine therapy. Omission should be considered in patients who are appropriate candidates for endocrine therapy and expected to tolerate and comply with treatment [11, 42]. Patients unlikely to initiate or adhere to endocrine therapy may warrant individualized consideration of nodal staging if results would meaningfully influence alternative management strategies.

## Implementation of Choosing Wisely^®^ Guideline to Omit SLNB in Women ≥ 70 Years Old

### De-implementation of SLNB in eligible women ≥ 70 years old

Several studies have examined national and institutional trends in de-implementation of SLNB among eligible women. Evidence of meaningful reductions in SLNB use has primarily come from single-institution academic centers, which have reported significant declines following publication of the long-term CALGB 9343 results and the SSO Choosing Wisely^®^ recommendation [52, 68, 71, 52–84]. In contrast, population-based analyses using SEER and NCDB data have generally shown minimal change, with persistently low rates of SLNB omission. However, these studies largely included patients diagnosed through 2017, shortly after release of the Choosing Wisely^®^ recommendation, potentially limiting their ability to capture evolving practice patterns [78, 85–88].

More recent NCDB data including patients diagnosed between 2017 and 2020 demonstrated gradual increases in SLNB omission over time; nevertheless, overall adherence remained low, with more than 75% of eligible women still undergoing SLNB [89]. Similar findings from single-institution studies show modest improvements in guideline concordance but continued high utilization of SLNB in patients meeting omission criteria [70, 90].

Conversely, some studies have found no measurable impact of the Choosing Wisely^®^ recommendation on clinical practice [66, 91]. Alarmingly, several analyses have even reported increasing SLNB use among women eligible for omission, despite randomized trial evidence and national guidelines supporting de-implementation [92–95]. Collectively, these findings highlight a persistent gap between evidence-based recommendations and real-world practice, suggesting the need for active de-implementation strategies to promote guideline-concordant care and reduce low-value axillary surgery in this population.

### Factors associated with increased omission of SLNB

Several patient-, tumor-, and provider-level factors have been associated with an increased likelihood of omitting SLNB in eligible women. Age consistently emerges as the strongest predictor, with omission rates increasingly progressively among older patients [52, 84, 86–89, 91–93, 96, 97]. Higher comorbidity burden [68, 92, 96] and greater clinical frailty [78, 87] are also strongly associated with omission, as is an estimated life expectancy of less than 5 years [78]. Tumor-specific characteristics, including smaller tumor size and lower histologic grade, have likewise been associated with higher omission rates, reflecting clinician willingness to de-escalate axillary staging in patients with more favorable disease biology [87, 89, 91].

Provider and institutional characteristics also significantly influence SLNB omission. Care delivered at academic or high-volume centers, or by surgeons with fellowship training in breast or surgical oncology, is associated with greater adherence to omission guidelines [88, 89, 94, 96]. These patterns may reflect increased familiarity with evolving evidence and greater comfort with de-escalation strategies, particularly among surgeons with specialized training or those earlier in their careers. Together, these findings suggest that both patient health status and provider expertise play critical roles in determining adherence to SLNB omission recommendations.

## Proposed Clinical Decision Algorithm for Safe Omission of SLNB in Elderly Women

**Figure 2:**
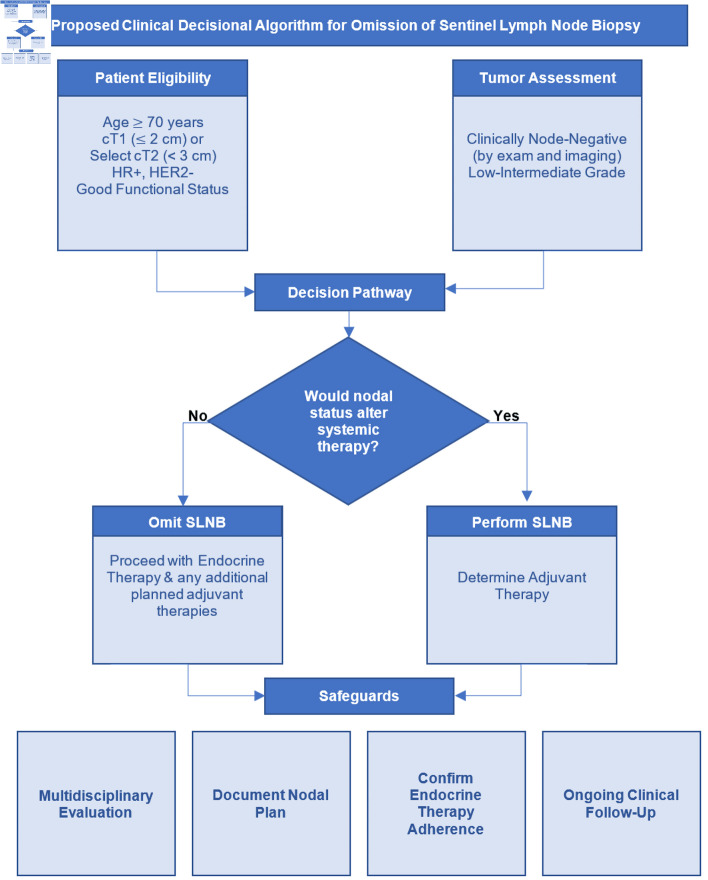
Clinical decisional algorithm for omission of sentinel lymph node biopsy (SLNB) in early-stage HR-positive, HER2-negative breast cancer. The algorithm outlines a multidisciplinary approach to identify patients, in whom SLNB may be safely omitted. The process begins with confirmation of clinically node-negative status and assessment of tumor biology and overall recurrence risk. The key decision point is whether nodal staging would alter adjuvant systemic or radiation therapy recommendations. In patients with favorable tumor biology, low anticipated benefit from chemotherapy, and a planned course of endocrine therapy, omission of SLNB is appropriate. Additional considerations include physiologic age, comorbidity burden, life expectancy, and treatment tolerance. Safe implementation requires shared decision-making, confirmation of endocrine therapy initiation, and routine clinical follow-up. This framework positions SLNB as a selective staging procedure rather than routine practice, aligning axillary management with contemporary biologically driven treatment paradigms. HR: hormone receptor; HER2: human epidermal growth factor receptor 2.

Based on the cumulative evidence discussed in this review, we propose a structured clinical framework to guide the safe omission of SLNB in carefully selected patients (Fig. 2). This approach translates existing data into a pragmatic, multidisciplinary pathway that integrates tumor biology, physiologic age, competing mortality risk, and endocrine therapy considerations.

Appropriate candidates include patients with clinically node-negative disease on physical examination and imaging, HR-positive/HER2-negative invasive breast cancer with otherwise favorable tumor features. The most robust data support omission in cT1 tumors; however, selected low-risk cT2 tumors (< 3 cm) may also be considered if no additional high-risk features are present. Although, it is important to keep in mind that nodal positivity increases with tumor size. Low-to intermediate-grade histology further strengthens eligibility. Although age ≥ 70 has been the most extensively studied within the context of the Choosing Wisely^®^ recommendations, this threshold should not be interpreted as a rigid chronological cutoff. Rather, age serves as a surrogate for competing mortality risk, physiologic reserve, comorbidity burden, treatment tolerance, and the probability that nodal information will meaningfully alter adjuvant therapy decisions. We therefore advocate for individualized assessment incorporating functional status, life expectancy, and tumor biology rather than chronological age alone. Importantly, patients should be appropriate candidates for, and likely to adhere to, endocrine therapy.

Implementation requires multidisciplinary evaluation and shared decision-making, including explicit confirmation that nodal status would not change planned management. The pathway begins with verification of clinical node-negative status and favorable tumor biology, followed by a focused determination of whether SLNB findings would influence systemic or radiation therapy. If nodal information is unlikely to modify treatment, omission is appropriate. Conversely, if axillary staging would guide therapeutic decision-making, SLNB should be performed.

In this context, SLNB functions as a selective staging tool rather than a routine intervention. Applied judiciously, omission represents an evidence-based refinement of axillary management that minimizes treatment-related morbidity without compromising oncologic outcomes.

## Conclusions

Management of the axilla in breast cancer has evolved from routine radical dissection to a selective, biologically informed approach. Robust evidence demonstrates that omission of SLNB in carefully selected postmenopausal women ≥ 70 years old with clinically node-negative, early stage, HR-positive/HER2-negative breast cancer is associated with very low rates of axillary recurrence and no clear detriment in DFS, BCSS, or OS, when appropriate endocrine therapy is administered. These findings led to the development of the Choosing Wisely^®^ recommendation and its subsequent endorsement by major professional societies.

Despite strong supporting data, adoption of SLNB omission remains inconsistent. Observational studies suggest that lower use of adjuvant therapy in omission cohorts often reflects patient age, comorbidity burden, and competing mortality risk rather than absence of nodal staging itself. Accordingly, de-escalation of axillary surgery should be implemented within a multidisciplinary framework that ensures appropriate endocrine therapy, individualized risk assessment, and shared decision-making.

As molecular profiling and risk stratification tools continue to refine prognostication, axillary management should remain tailored to tumor biology and health status. When nodal information is unlikely to influence systemic treatment decisions, omission of SLNB represents a safe, evidence-based refinement of surgical care that reduces morbidity while preserving oncologic outcomes in older women with biologically favorable breast cancer.

## Supplementary Material

Suppl 1 Landmark randomized controlled trials providing historical context: de-escalation as a foundation for omission of axillary surgery in breast cancer.

Suppl 2 Evidence discussing omission of axillary surgery in postmenopausal women.

## Data Availability

The authors declare that data supporting the findings of this study are available within the article.

## Abbreviations

ALND: axillary lymph node dissection
SLNB: sentinel lymph node biopsy
HR: hormone receptor
HER2: human epidermal growth factor receptor 2
cN0: clinically node-negative
NSABP: National Surgical Adjuvant Breast and Bowel Project
BCS: breast conserving surgery
DFS: disease-free survival
RFS: recurrence-free survival
DDFS: distant disease-free survival
OS: overall survival
IBCSG: International Breast Cancer Study Group
cALND: completion axillary lymph node dissection
AMAROS: After Mapping of the Axilla, Radiotherapy or Surgery
OTOASOR: Optimal Treatment of the Axilla- Surgery Or Radiotherapy
BMI: body mass index
ALMANAC: Axillary-Lymphatic Mapping Against Nodal Axillary Clearance
ABIM: American Board of Internal Medicine
SOUND: Sentinel Node vs. Observation After Axillary Ultra-Sound
INSEMA: Intergroup Sentinel Mamma
LRFS: locoregional recurrence-free survival
CALGB: Cancer and Leukemia Group B
BCSS: breast cancer-specific survival
ART: axillary radiotherapy
SSO: Society of Surgical Oncology
NCCN: National Comprehensive Cancer Network
ER: estrogen receptor
SEER: Surveillance, Epidemiology, and End Results Program
NCDB: National Cancer Database
OR: odds ratio
CI: confidence interval
RR: relative risk
