# Supplementary material for: De-Escalation of Axillary Surgery: A Review of Choosing Wisely Guideline Evidence
Source: World J Oncol. 2026 May 8;17(3):277–91. doi: 10.14740/wjon2710 (PMC13171263; doi:10.14740/wjon2710)
Supplement: Suppl 1 — Landmark randomized controlled trials providing historical context: de-escalation as a foundation for omission of axillary surgery in breast cancer. [file wjon-17-03-277-s001.docx]

| **Suppl 1. Landmark Randomized Controlled Trials Providing Historical Context: De-escalation as a Foundation for Omission of Axillary Surgery in Breast Cancer** | | | | | | | | | |
| --- | --- | --- | --- | --- | --- | --- | --- | --- | --- |
| **Trial/study, author** | **Enrollment years** | **Follow-up** | **Key Question** | **Population** | **Sample size** | **Recurrence** | **Survival** | **Key findings** | **Limitations** |
| **NSABP B-04,** Fisher et al.  (2002) [1] | 1971-1974 | 25 years | Does radical mastectomy improve outcomes compared with less extensive surgery $\pm$ radiation? | Women with operable breast cancer   - cN0   - Radical mastectomy   - Total mastectomy + ALND + regional RT   - Total mastectomy alone - cN+   - Halstead radical mastectomy   - Total mastectomy + regional RT | *n* = 1665 | cN0   - Radical mastectomy:   - Local: 5.2%   - Regional: 4.1%   - Distant: 27.9% - Total mastectomy + ALND + regional RT:   - Local: 1.42%   - Regional: 4.3%   - Distant: 31.5% - Total mastectomy alone:   - Local: 7.1%   - Regional: 6.3%   - Distant: 29.3%   cN+   - Radical mastectomy:   - Local: 7.9%   - Regional: 7.5%   - Distant: 41.1% - Total mastectomy + regional RT:   - Local: 2.7%   - Regional: 11.2%   - Distant: 43.2% | No significant differences in DFS, RFS, DDFS, or OS among women in the cN0 groups or the cN+ groups  25-year results  cN0   - Radical mastectomy: DFS 19 $\pm$2%, RFS 53 $\pm$ 3%, DDFS 46 $\pm$ 3%; OS 25 $\pm$ 3% - Total mastectomy + ALND + regional RT: DFS 13 $\pm$ 2% (HR 1.06; 95% CI 0.90-1.25; p=0.49), RFS 52 $\pm$ 4% (HR 0.96; 95% CI 0.76-1.21; p=0.74), DDFS 38 $\pm$ 3% (HR 1.08; 95% CI 0.88-1.34; p=0.44); OS 19 $\pm$ 2% (HR 1.08; 95% CI 0.91-1.28; p=0.38) - Total mastectomy alone: DFS 19 $\pm$ 2% (HR 1.07; 95% CI 0.91-1.27; p=0.39), RFS 50 $\pm$ 3% (HR 1.14; 95% CI 0.91-1.42; p=0.27), DDFS 43 $\pm$ 3% (HR 1.10; 95% CI 0.89-1.35; p=0.39); OS 26 $\pm$ 3% (HR 1.03; 95% CI 0.87-1.23, p=0.72)   cN+   - Radical mastectomy: DFS 11 $\pm$ 2%, RFS 36 $\pm$ 3%; DDFS 32 $\pm$ 3%; OS 14 $\pm$ 2% - Total mastectomy + regional RT: DFS 10 $\pm$2% (HR 1.12; 95% CI, 0.94-1.33; p=0.20), 33 $\pm$ 3% (HR 1.09; 95% CI 0.89-1.35; p=0.40); DDFS 29 $\pm$ 3% (HR 1.07; 95% CI 0.87-1.32; p=0.51); OS 14 $\pm$ 2% (HR 1.06; 95% CI 0.89-1.27; p=0.49)   Statistical analysis via log-rank tests, cox proportional-hazards models | No survival advantage from radical mastectomy or removal of occult positive nodes | Conducted before modern systemic therapies, lacks stratification by tumor biomarkers, lack of subgroup analysis, and dated surgical and radiation techniques limit relevance to modern practice |
| **NSABP B-32**  Krag et al.  (2010) [2] | 1999-2004 | 8 years | Is SLNB alone safe in node negative patients? | Women with cT1-3, cN0 invasive breast cancer, undergoing BCS or mastectomy, randomized to   - SLNB + ALND - SLNB with ALND only if SLNs + | *n* = 5611 | No significant difference in regional control   - SLNB + ALND   - Local: 2.7%   - Regional: 0.4%   - Distant: 2.8% - SLNB   - Local: 2.4%   - Regional: 0.7%   - Distant: 3.2%   Regional recurrence p=0.22  Statistical analysis via cumulative incidence curves, cause specific hazard rates | No significant difference in OS and DFS   - SLNB + ALND: OS 91.8% (95% CI 90.4-93.3), DFS 89.0% (95% CI 87.6-90.4) - SLNB: OS 90.3% (95% CI 88.8-91.8); DFS 81.5% (95% CI 79.6-83.4)   OS unadjusted HR of 1.20 (95% CI 0.96-1.50; p=0.12); adjusted HR 1.19; 95% CI 0.95-1.49; p=0.13)  DFS unadjusted HR of 1.05 (95% CI 0.90-1.22; p=0.54); adjusted HR was 1.07 (95% CI 0.90-1.22; p=0.57)  Statistical analysis via log-rank comparison, cox proportional hazard analyses, Kaplan-Meier method | No significant survival advantage between SLNB followed by ALND and SLNB alone in node negative patients | Conducted before modern systemic therapy, limited to cN0 population, does not apply to neoadjuvant setting, non-standardized nodal radiation |
| **IBCSG 23-01**  Galimberti et al. (2018) [3] | 2001-2010 | 10 years | Can ALND be omitted in patients with SLN micro-metastases? | Women with cT1-2 ($\leq$ 5 cm), cN0 breast cancer with 1 or more micro-metastases in the SLN undergoing BCS or mastectomy, randomized to   - cALND - No ALND | *n* = 934 | - cALND   - Local: 3%   - Regional: 1%   - Distant: 10% - No ALND   - Local: 3%   - Regional: 2%   - Distant: 9% | No significant difference in DFS or OS   - cALND: DFS 74.9% (HR 0.85, 95% CI 70.5-79.3) log-rank p=0.24; p=0.0024 non-inferiority, OS 88.2% (HR 0.78; 95% CI 0.53-1.14) log-rank p=0.20 - No ALND: DFS 76.8% (95% CI 72.5-81.0), OS 90.8% (95% CI 87.9-93.8)   Statistical analysis via log-rank comparison | SLNB alone is noninferior to ALND in the micro-metastatic setting | Underpowered due to early closure, predominantly included low risk, small HR+ tumors |
| **AATRM 048**  Solá et al. (2013) [4] | 2001-2008 | 5 years | Can ALND be omitted in patients with SLN micro-metastases? | Women with early stage breast cancer (T < 3.5 cm, cN0, M0) with SLN micro-metastases in the SLN, undergoing BCS or mastectomy, randomized to   - cALND - No ALND | *n* = 233 | - cALND   - Local: 0%   - Regional: 0.9%   - Distant: 0% - No ALND   - Local: 0%   - Regional: 1.6%   - Distant: 0.8% | No significant difference in DFS (p=0.330)  Statistical analysis by Kaplan-Meier method, log-rank test | SLNB alone offers adequate locoregional and distant disease control without impact on survival in the micro-metastatic setting | Small sample size, underpowered, low recurrence event rates, limited follow up |
| **ACOSOG Z0011**  Giuliano et al. (2011, 2017) [5–7] | 1999-2004 | 10 years | Can ALND be omitted in 1-2 positive SLNs? | Women with cT1-T2, cN0 breast cancer without palpable axillary lymphadenopathy undergoing lumpectomy, whole breast radiation, and adjuvant systemic therapy found to have 1-2 positive SLNs (micro & macro-metastases), randomized to   - cALND - No ALND | *n* = 891 | - cALND   - Local: 5.6%   - Regional: 0.5% - No ALND   - Local: 3.8%   - Regional: 1.5%   Cumulative incidence of locoregional recurrence did not differ between each study arm (p=0.36)  Statistical analysis via Kaplan-Meier method and log-rank test | No significant difference in OS   - cALND: OS 83.6% (95% CI 79.1-87.1), DFS 78.2% (95% CI 73.5-82.2%) - No ALND: OS 86.3% (95% CI 82.2-89.5), DFS 80.2% (95% CI 75.6-84.1)   SLNB alone was noninferior for OS (log-rank p=0.02); DFS (log-rank p=0.32)  Unadjusted HR comparing OS between ALND vs. no ALND: 0.85 (1-sided 95% CI 0-1.16), did not cross the pre-specified noninferiority margin HR of 1.3  Unadjusted HR comparing DFS between ALND vs. no ALND: 0.85 (95% CI 0.62-1.17) | ALND can be safely omitted in patients with 1-2 positive SLNs undergoing BCS with WBI and systemic therapy; practice-changing for axillary management;  SLNB alone is non-inferior to ALND for OS | Underpowered, excluded mastectomy, incidental axillary RT due to lack of standardized RT fields, high rates of systemic therapy |
| **SINODAR-ONE**  Tinterri et al. (2022) [8] | 2015-2020 | 4 years | Can ALND be omitted in 1-2 SLN macro-metastases? | Women with cT1-T2, cN0 breast cancer, clinically node negative by US, undergoing BCS with radiation or mastectomy with 1-2 macro-metastatic SLNs, randomized to   - cALND - No ALND | *n* = 889 | - cALND   - Local: 0%   - Regional: 0.2%   - Distant: 1.7% - No ALND   - Local: 0.7%   - Regional: 0.2%   - Distant: 1.9%   Cumulative incidence of local, regional, and distant recurrence did not differ between each study arm (p=0.444) | No significant difference in OS or RFS   - cALND: OS 98.9%, RFS 96.3% - No ALND: OS 98.8%, RFS 95.6%   Statistical analysis by Kaplan-Meier method, log-rank test | SLNB alone is noninferior to ALND in patients with 1-2 macro-metastatic SLNs; includes mastectomy patients | Limited follow up duration, predominantly low risk patients, few mastectomy patients, low event rates; primary OS endpoint not yet mature |
| **SENOMAC**  De Boniface et al. (2024) [9] | 2015-2021 | 5 years | Can ALND be omitted in patients with 1-2 SLN macro-metastases? | Women and male patients with cT1-3, cN0 by US breast cancer with 1-2 SLN macro-metastases, extracapsular extension allowed, undergoing BCS with radiation or mastectomy, randomized to   - cALND - No ALND   Patients undergoing neoadjuvant systemic therapy were eligible if SLNB was performed before start of treatment | *n* = 2766 | - cALND   - Local: 0.8%   - Regional: 0.5%   - Distant: 4.4% - No ALND   - Local: 0.9%   - Regional: 0.4%   - Distant: 3.3% | No significant difference in OS or RFS   - cALND: OS 92.0% (95% CI 89.9-94.1), BCSS 96.6% (95% CI 95.3-97.9), RFS 88.7% (95% CI 86.3-91.1) - No ALND: OS 92.9% (95% CI 91.0-94.9), BCSS 97.1% (95% CI 95.8-98.3), RFS 89.7% (95% CI 87.5-91.9)   HR for recurrence or death in the No ALND group compared to cALND group was 0.89 (95% CI 0.66-1.19; p=<0.001) below the noninferiority margin  Statistical analysis by Kaplan-Meier method, Cox proportional-hazards model | SLNB alone is noninferior to ALND in clinically node negative, T1-T3 breast cancer with SLN macro-metastasis with extracapsular extension, receiving systemic treatment and RT per national guidelines | Limited follow up duration, higher rates of nodal field irradiation, predominantly low risk tumors, low event rates |
| **SERC**  Houvenaeghel et al. (2021) [10] | 2012-2021 | Ongoing | Can ALND be omitted for patients with SLN involvement (isolated tumor cells, micro, or macro-metastases)? | Patients with cT0-2, cN0 patients undergoing BCS or mastectomy with isolated tumor cells, micro, or macro-metastases identified on SLNB, randomized to   - cALND - No ALND | *n* = 1855 | Primary recurrence outcomes not yet published | Primary survival outcomes not yet published | Ongoing | Ongoing |
| **OTOASOR**  Sávolt et al. (2017) [11] | 2002-2009 | 8 years | Does axillary RT provide equivalent regional control to ALND in SLN-positive (pN1) patients? | Women with primary invasive cT $\leq$ 3 cm, cN0 by US undergoing BCS with radiation or mastectomy with SLNB, randomized to   - cALND - RNI (Whole breast + all 3 levels of the axilla and supraclavicular fossa, 50 Gy) | *n* = 474 | - cALND   - Regional: 2.0% - RNI:   - Regional: 1.7%   p=1.00  Statistical incidence via cumulative incidence method | No significant difference in OS or DFS   - cALND: OS 77.9%, DFS 72.1% - RNI: OS 84.8%, DFS 77.4%   OS p=0.060, HR 0.59  DFS p=0.51  Statistical analysis by Cox proportional hazards regression and Kaplan-Meier method | RNI is non-inferior to cALND and is an alternative treatment strategy for select patients | Single center study, small sample size, predominantly low risk tumors, low event rate, few mastectomy patients |
| **AMAROS**  Bartels et al. (2023) [12] | 2001-2010 | 10 years | Does axillary RT provide equivalent regional control to ALND in SLN-positive patients? | Women with cT1-2, cN0 undergoing BCS or mastectomy, with positive SLNB, randomized to   - ALND - Axillary radiotherapy (ART) (all 3 levels of the axilla and medial part of the supraclavicular fossa, 50 Gy) | *n* = 1425 | - ALND   - Local: 2.5%   - Regional: 0.9%   - Distant: 12.0%   - Second primary cancers cumulative incidence: 8.3% (95% CI 6.3-10.7)   - Axillary Recurrence rate (ARR) cumulative incidence: 0.93% (95% CI 0.18-1.68) - Axillary radiotherapy   - Local: 2.2%   - Regional: 1.6%   - Distant: 14.5%   - Second primary cancers cumulative incidence: 12.1% (95% CI 9.6-14.9), (HR 1.45; 95% CI 1.03-2.04; p=0.035)   - ARR cumulative incidence:1.82% (95% CI 0.74-2.94), (HR 1.71; 95% CI 0.67-4.39) | No significant difference in OS or DFS   - ALND: OS 84.6% (95% CI 81.5-87.1), DFS 75.0% (95% CI 71.5-78.2) - ART: OS 81.4% (95% CI 77.9-84.4), DFS 70.1% (95% CI 66.2-73.6)   OS (HR 1.17; 95% CI 0.89-1.52; p=0.26)  DFS (HR 1.19; 95% CI 0.97-1.46; p=0.11)  Statistical analysis by Kaplan-Meier method | Axillary radiotherapy is a safe alternative to cALND in this select population, with lower rates of lymphedema, although has a higher risk of secondary primary cancer near the radiation field | Predominantly low risk tumors, few mastectomy patients |

[1] Fisher B, Jeong JH, Anderson S, Bryant J, Fisher ER, Wolmark N: Twenty-five-year follow-up of a randomized trial comparing radical mastectomy, total mastectomy, and total mastectomy followed by irradiation. N Engl J Med 2002, 347:567-575.

[2] Krag DN, Anderson SJ, Julian TB, Brown AM, Harlow SP, Costantino JP, Ashikaga T, Weaver DL, Mamounas EP, Jalovec LM, et al: Sentinel-lymph-node resection compared with conventional axillary-lymph-node dissection in clinically node-negative patients with breast cancer: overall survival findings from the NSABP B-32 randomised phase 3 trial. Lancet Oncol 2010, 11:927-933.

[3] Galimberti V, Cole BF, Viale G, Veronesi P, Vicini E, Intra M, Mazzarol G, Massarut S, Zgajnar J, Taffurelli M, et al: Axillary dissection versus no axillary dissection in patients with breast cancer and sentinel-node micrometastases (IBCSG 23-01): 10-year follow-up of a randomised, controlled phase 3 trial. Lancet Oncol 2018, 19:1385-1393.

[4] Sola M, Alberro JA, Fraile M, Santesteban P, Ramos M, Fabregas R, Moral A, Ballester B, Vidal S: Complete axillary lymph node dissection versus clinical follow-up in breast cancer patients with sentinel node micrometastasis: final results from the multicenter clinical trial AATRM 048/13/2000. Ann Surg Oncol 2013, 20:120-127.

[5] Giuliano AE, Ballman KV, McCall L, Beitsch PD, Brennan MB, Kelemen PR, Ollila DW, Hansen NM, Whitworth PW, Blumencranz PW, et al: Effect of Axillary Dissection vs No Axillary Dissection on 10-Year Overall Survival Among Women With Invasive Breast Cancer and Sentinel Node Metastasis: The ACOSOG Z0011 (Alliance) Randomized Clinical Trial. JAMA 2017, 318:918-926.

[6] Giuliano AE, Hunt KK, Ballman KV, Beitsch PD, Whitworth PW, Blumencranz PW, Leitch AM, Saha S, McCall LM, Morrow M: Axillary dissection vs no axillary dissection in women with invasive breast cancer and sentinel node metastasis: a randomized clinical trial. JAMA 2011, 305:569-575.

[7] Giuliano AE, Ballman K, McCall L, Beitsch P, Whitworth PW, Blumencranz P, Leitch AM, Saha S, Morrow M, Hunt KK: Locoregional Recurrence After Sentinel Lymph Node Dissection With or Without Axillary Dissection in Patients With Sentinel Lymph Node Metastases: Long-term Follow-up From the American College of Surgeons Oncology Group (Alliance) ACOSOG Z0011 Randomized Trial. Ann Surg 2016, 264:413-420.

[8] Tinterri C, Gentile D, Gatzemeier W, Sagona A, Barbieri E, Testori A, Errico V, Bottini A, Marrazzo E, Dani C, et al: Preservation of Axillary Lymph Nodes Compared with Complete Dissection in T1-2 Breast Cancer Patients Presenting One or Two Metastatic Sentinel Lymph Nodes: The SINODAR-ONE Multicenter Randomized Clinical Trial. Ann Surg Oncol 2022, 29:5732-5744.

[9] de Boniface J, Filtenborg Tvedskov T, Ryden L, Szulkin R, Reimer T, Kuhn T, Kontos M, Gentilini OD, Olofsson Bagge R, Sund M, et al: Omitting Axillary Dissection in Breast Cancer with Sentinel-Node Metastases. N Engl J Med 2024, 390:1163-1175.

[10] Houvenaeghel G, Cohen M, Raro P, De Troyer J, Gimbergues P, Tunon de Lara C, Ceccato V, Vaini-Cowen V, Faure-Virelizier C, Marchal F, et al: Sentinel node involvement with or without completion axillary lymph node dissection: treatment and pathologic results of randomized SERC trial. NPJ Breast Cancer 2021, 7:133.

[11] Savolt A, Peley G, Polgar C, Udvarhelyi N, Rubovszky G, Kovacs E, Gyorffy B, Kasler M, Matrai Z: Eight-year follow up result of the OTOASOR trial: The Optimal Treatment Of the Axilla - Surgery Or Radiotherapy after positive sentinel lymph node biopsy in early-stage breast cancer: A randomized, single centre, phase III, non-inferiority trial. Eur J Surg Oncol 2017, 43:672-679.

[12] Bartels SAL, Donker M, Poncet C, Sauve N, Straver ME, van de Velde CJH, Mansel RE, Blanken C, Orzalesi L, Klinkenbijl JHG, et al: Radiotherapy or Surgery of the Axilla After a Positive Sentinel Node in Breast Cancer: 10-Year Results of the Randomized Controlled EORTC 10981-22023 AMAROS Trial. J Clin Oncol 2023, 41:2159-2165.

| **Suppl 2. Evidence Discussing Omission of Axillary Surgery in Postmenopausal Women** | | | | | | | | | | | |
| --- | --- | --- | --- | --- | --- | --- | --- | --- | --- | --- | --- |
| **Design** | **Enrollment Years** | **Follow Up** | **Key Question** | **Population** | **Tumor markers** | **Sample Size** | **Survival** | **Recurrence** | **Key Conclusions** | **Limitations** |  |
| **Randomized Controlled Trials (RCT)** | | | | | | | | | | | |
| RCT | 1994-1999 | 12 years | What is the benefit of adjuvant radiation after BCS? | Women $\geq$ 70 years old with cT1N0M0, ER+ breast cancer undergoing lumpectomy, randomized to   - Tamoxifen + RT (TamRT) - Tamoxifen (Tam)   ALND was left to the surgeon’s discretion | ER+ | *n* = 636 | No significant difference in OS or BCSS   - TamRT: OS 67% (95% CI 62-72), BCSS 97% (95% CI 94-99%) - Tam: OS 66% (95% CI 61-71), BCSS 98% (95% CI 95-99)   Statistical analysis by Kaplan-Meier method and log-rank | - TamRT:   - Local: 1.9%   - Regional: 0%   - Distant: 6.6% - Tam:   - Local: 6.3%   - Regional: 1.9%   - Distant: 5.0% | Omitting adjuvant RT results in increased locoregional recurrence without impacting survival outcomes; incidentally identified low rates of axillary recurrence in patients where ALND was omitted, raising the possibility that ALND may be omitted in elderly women with stage I, ER+ breast cancers | Not designed to study impact of omitting axillary staging on recurrence and survival outcomes, lacked the power to demonstrate non-inferiority |  |
| RCT | 1996-2000 | 15 years | Is it safe to omit axillary surgery in elderly women with early breast cancer who are clinically node negative? | Women 65-80 years old with cT1N0 breast cancer, who underwent BCS + RT, were randomized to   - ALND - No ALND   All patients were prescribed tamoxifen for 5 years | Predom.ER+, PR+ | *n* = 238 | No significant difference in OS or breast cancer mortality   - ALND:   - Breast cancer mortality: 7.6% (95% CI 2.5-12.7%) - No ALND:   - Breast cancer mortality: 9.2% (95% CI 3.7-14.6%)   Statistical analysis by Kaplan-Meier method, multivariable Fine and Gray Models | 15-year Crude Cumulative Incidence of Recurrence   - ALND   - Local: 4% (95% CI 0.1-7.8%)   - Axillary: 0%   - Distant: 8.6% (95% CI 3.2-13.9%) - No ALND   - Local: 8.3% (95% CI 2.1-14.5%)   - Axillary: 6%   - Distant: 9.6% (95% CI 3.3-15.9%)   Statistical analysis by Gray test | No significant difference OS and breast cancer mortality between patients undergoing vs omitting ALND, supports omission of axillary surgery in this patient population treated with BCS, adjuvant RT, and adjuvant tamoxifen | Study was underpowered to demonstrate non-inferiority, small sample size |  |
| RCT | 1993-2002 | 6 years | Does omitting ALND lead to improved quality of life without impacting survival outcomes? | Women $\geq$ 60 years old with cT1-3N0M0 operable breast cancer undergoing breast surgery (BCS with adjuvant RT or mastectomy) with adjuvant tamoxifen therapy for 5 years, randomized to   - ALND - No ALND | Predom. ER+ | *n* = 473 | No significant difference in DFS or OS   - ALND: DFS 67%, OS 75% - No ALND: DFS 66% (HR 1.06; 95% CI 0.79-1.42; p=0.69), OS 73% (HR 1.05; 95% CI 0.76-1.46; p=0.77)   Statistical analysis by Kaplan-Meier method | No significant difference in axillary recurrence between groups was found   - ALND:   - Local: 4%   - Axillary: 1%   - Distant: 12% - No ALND   - Local: 2%   - Axillary: 3%   - Distant: 10% | Omitting ALND does not compromise DFS or OS and is associated with transient improved quality of life in the immediate post-op setting | Study was small with short term follow up, underpowered for analysis of survival outcomes and transitioned focus to quality of life outcomes |  |
| **Single Institution Studies** | | | | | | | | | | | |
| **Design** | **Enrollment Years** | **Follow Up** | **Key Question** | **Population** | **Tumor markers** | **Sample Size** | **Survival** | **Recurrence** | **Key Conclusions** | **Limitations** |  |
| Retrospective cohort | 1987-1992 | 15 years | Is omitting axillary surgery safe in the long term for elderly patients with cN0 breast cancer? | Women $\geq$ 70 years old with cN0 invasive breast cancer undergoing BCS with or without ALND; adjuvant RT & decision regarding ALND per treating physician; prescribed tamoxifen for at least 2 years regardless of hormone status   - ALND - No ALND | Predom. ER/PR+ | *n* = 671 | No significant difference in 15-year crude cumulative incidence estimate of breast cancer mortality (p=0.657)   - ALND: 13.6% (95% CI 9.2-19.9%) - No ALND: 14.0% (95% CI 11.2-17.4%)   Statistical analysis by Fine and Gray model | 15-year crude cumulative incidence estimates:   - ALND - Local: 7.7% - Axillary: 0% - Distant: 11.6% - No ALND - Local: 6.3% - Axillary: 5.8% - Distant: 9.9%   Statistical analysis by Gray test | No benefit in breast cancer mortality for undergoing ALND in cN0 elderly patients with early breast cancer, suggests SLNB could also be omitted | Single institution study, low rates of adjuvant breast RT, limited survival outcome analyses |  |
| Retrospective cohort | 2000-2011 | 5 years | Is it safe to omit SLNB in patients over 70 years old with cN0 breast cancer? | Women $\geq$ 70 years old with cT1-2N0 invasive breast cancer undergoing BCS without SLNB | Predom. ER/PR+ | *n* = 140 | 5-year OS 70%  5-year BCSS 96% | 4% experienced breast cancer related event:  1 axillary recurrence  4 deaths | Omitting SLNB in this population limits morbidity in patients more likely to die from their comorbidities | Small, single institution study, low rates of hormone & adjuvant RT |  |
| Retrospective cohort | 1995-2006 | 10 years | How does omitting axillary surgery in low risk women impact axillary recurrence and survival outcomes? | Low risk women (post-menopausal, ER+, invasive breast cancers <20 mm grade 1 or <15 mm grade 2, without lymphovascular invasion) who underwent BCS with whole breast radiation or mastectomy, receiving adjuvant endocrine therapy did not undergo axillary surgery | ER+ | *n* = 194 | OS:  5-years 90.3% (95% CI 83.6-94.4%)  10-years 75.5% (95% CI 65.9-82.8%)  DFS:  5-years 96.6% (95% CI 91.1-98.7%)  10-years 91.2% (95% CI 82.6-96.6%)  DDFS:  5-years 99.2% (95% CI 94.1-99.9%)  10-years 97% (95% CI 90.0-99%)  Statistical analysis by Kaplan- Meier method | Axillary recurrence cumulative incidence: 0.8% at 5 years  1.9% at 10 years | Low risk women (post-menopausal with tumors <20 mm grade 1 or <15 mm grade 2, ER+, and lymphovascular invasion -) may omit axillary surgery with low risk of axillary recurrence | Single center study, low rates of mastectomy, study design |  |
| Retrospective cohort | 1994-2008 | 10 years | How does omitting axillary staging in elderly women impact recurrence and survival? | Women $\geq$ 70 years old with primary invasive breast cancer operated on at the European Institute of Oncology between 1994-2008 were followed. Axillary surgery (SLNB or ALND) was omitted due to age >80 at diagnosis, cN0, early stage, significant comorbidities, favorable prognostic factors on biopsy. Matched analyses were performed comparing axillary surgery vs no axillary surgery. | Luminal A and B | *n*  = 1748 | No significant difference on OS at 5 and 10 years (p=0.52)  Overall survival:   - Axillary surgery: 87.9% at 5 years, 69.1% at 10 years - No axillary surgery: 85.3% at 5 years, 69.0% at 10 years   Statistical analysis by Kaplan-Meier method and log-rank test | Significant increased risk of developing axillary recurrence in the no axillary surgery group at 5- and 10-year end points (p=0.038)   - Axillary surgery - Local: 10.6% - Regional: 1.7% - Distant: 7.1% - No axillary surgery - Local: 10.7% - Regional: 8.9%* - Distant: 10.8%   Statistical analysis by Gray’s test | Omission of axillary surgery led to increased risk of axillary recurrence, although this failed to significantly impact OS indicating feasibility to omit axillary staging in elderly women | Single institution study, selection bias, data missing from database regarding tumor biomarkers, adjuvant therapy specifics |  |
| Prospective, observational | 2016-2022 | 3 years | What is the impact of omitting SLNB on axillary recurrence and survival in elderly patients with low risk cancers? | Women $\geq$ 65 years old with cT1-2N0 ER+, HER2- invasive breast cancer undergoing BCS without SLNB. Patients were planned to undergo adjuvant endocrine therapy with or without RT. Axilla was examined with US preoperatively. | ER+, HER2- | *n* = 125 | 3-year rates of  Regional RFS: 98.2%  DFS: 91.2%  BCSS: 99.2%  OS: 94.8%  Statistical analysis by Kaplan-Meier method | 3-year recurrence rates:  Local: 4.8%  Axillary: 1.6%  Distant: 1.6%  Hormonal therapy noncompliance associated with recurrence (p=0.02) by univariate cox regression analysis | In women $\geq$ 65 years old with cT1-2N0 ER+, HER2- invasive breast cancer omitting SLNB was not associated with a significant impact on survival or recurrence outcomes.  Endocrine therapy noncompliance was associated with increased recurrence | Did not reach targeted accrual, single institution, short follow up, low rate of endocrine compliance, low rates of adjuvant RT warrants longer term follow up to determine impact |  |
| Retrospective | 2014-2022 | 3 years | What are the trends, factors, and outcomes associated with omitting axillary staging in elderly breast cancer patients? | Women $\geq$ 70 years old with pT1, cN0, ER+/HER2- invasive breast cancer undergoing surgery 2014-2022 at the Mayo Clinic AZ, groups split into 2014-2016 & 2017-2022 for analysis pre- and post- CW guideline in addition to if the received or omitted axillary surgery. | ER+, HER2- | *n* = 218 | No significant difference between the groups in terms or LRFS (HR 0.60; 95% CI 0.10-3.61; p=0.57) or DFS (HR 1.23; 95% CI 0.49-3.07; p=0.66) for those who received vs omitted axillary surgery  Statistical analysis by Kaplan-Meier method, groups compared by log-rank test | - Axillary Surgery - Local: 2.5% - Axillary: 0% - Distant: 0% - No Axillary Surgery - Local: 2.0% - Axillary: 0% - Distant: 1% | In women $\geq$ 70 years old with pT1, cN0, ER+/HER2- breast cancer omitting SLNB did not impact survival or recurrence outcomes. Omission of both SLNB and RT rates are increasing since CW publication. | Single center study, short follow up, small sample size |  |
| Retrospective | 2016-2021 | 4 years | What is the impact of omitting SLNB and RT in elderly patients with early stage, favorable breast cancers? | Women $\geq$ 70 years old with cT1N0, HR+, HER2- breast cancer undergoing BCS without SLNB and RT at Ichilov Medical Center. Adjuvant endocrine therapy was recommended. | HR+, HER2- | *n* = 100 | Median DFS 42 months (11-128)  Tumor size > 13 mm associated with worse DFS (HR 4.02; 95% CI 1.08-14.99; p=0.04)  Statistical analysis by Kaplan-Meier method and cox proportional hazard regression model | 4-year recurrence rates:  Local: 4%  Locoregional: 2%  Distant: 1% | Omission of both SLNB and adjuvant RT is safe in early HR+, HER2- elderly breast cancer patients with small tumors | Single center, short term follow up, small sample size |  |
| **Registry Studies** | | | | | | | | | | | |
| **Design** | **Enrollment Years** | **Follow Up** | **Key Question** | **Population** | **Tumor markers** | **Sample Size** | **Survival** | **Recurrence** | **Key Conclusions** | **Limitations** |  |
| Retrospective cohort | 2001-2008 | 10 years | What is the impact of incomplete axillary staging on recurrence and survival outcomes? | Dutch women $\geq$ 75 years old with breast cancer undergoing BCS or mastectomy and SLNB, divided into two groups, using Netherlands Cancer Registry   - Complete Axillary Staging - Incomplete Axillary Staging | Predom. ER/PR+ | *n* = 1467 | No significant difference in OS (HR 1.21; 95% CI 0.96-1.53; p-log rank=0.101)   - Complete Axillary Staging: 32.7% (95% CI 30.5-35.4) - Incomplete Axillary Staging: OS 28.5% (95% CI 18.9-39.5)   Statistical analysis by Kaplan-Meier method, and log-rank | - Incomplete Axillary Staging - Local: 4.3% - Regional: 4.3% - Distant: 8.6%   Recurrence in complete axillary staging group not reported | In elderly breast cancer patients with more than 2 comorbidities, omission of complete axillary staging does not significantly impact regional disease control or OS | Small group of incomplete axillary staging, missing data (RT & HR status) for control group comparison |  |
| Population based cohort | 2004-2014 | 10 years | How does omitting axillary surgery impact survival? | Women $\geq$ 70 years old with cT1-3N0 invasive breast cancer in the National Cancer Database, using propensity score matching, undergoing   - Axillary Surgery - No Axillary Surgery | Predom. ER+, PR+ | *n* = 133,778 | Omitting Axillary Surgery was associated with worse OS on adjusted analysis (HR 1.66, 95% CI 1.61-1.70)   - Axillary Surgery: Unadjusted OS 111 months - No Axillary Surgery: Unadjusted OS 74.5 months   Unadjusted median OS (log-rank p<0.001): 111 months vs 74.5 months in those undergoing axillary surgery vs omitting  Adjusted OS: omitting nodal surgery associated with worse OS (HR 1.66, 95% CI 1.61-1.70, p<0.001)  Subgroup analysis cT1, grade 1 and 2, ER+ unadjusted median OS:  121.5 months vs 85.7 months (log-rank p<0.001) in those undergoing axillary surgery vs omitting  Statistical analysis by Kaplan-Meier method, log-rank test, Cox Proportional Hazards model | N/A | Undergoing axillary surgery was associated with improved OS | Limitations of NCDB data entry/coding, lack of BCSS data, lack of recurrence data, lack of complete HER2 data |  |
| Retrospective cohort | 2010-2018 | 5 years | What is the impact of omitting SLNB on axillary recurrence and survival? | Women $\geq$ 70 years old with ER+, HER2- cN0 invasive breast cancer were identified in the UPMC Network Cancer Registry, rates of SLNB and RT were examined along with survival and recurrence outcomes   - SLNB - No SLNB | ER+, HER2- | *n* = 2109 | No significant difference in LRFS or DFS   - LRFS (HR 1.26; 95% CI 0.37-4.30; p=0.71) - DFS (HR 1.92; 95% CI 0.86-4.32; p=0.11)   Statistical analysis by Kaplan-Meier method and log-rank test, multivariable Cox proportional hazards regression modeling | - No SLNB: - Local:1.2% - Distant: 2.5%   Absolute rates of recurrence:   - SLNB: 3.5% - No SLNB: 4.5% | Undergoing SLNB did not improve survival outcomes in ER+, HER2- cN0 elderly breast cancer patients | Single healthcare system registry data, limited comorbidity data potential for confounding, limited follow up |  |
| Retrospective cohort | 2005-2014 | 5 years | Is there a difference in survival in cN0 elderly patients who undergo axillary staging vs those who do not? | Korean women $\geq$ 70 years old with primary invasive breast cancer, cN0, undergoing BCS or mastectomy, divided into two groups using the Korean Breast Cancer Registry   - Axillary Surgery - No Axillary Surgery | Predom. ER+, HER2- | *n* = 2995 | No significant difference in OS or BCSS   - Axillary Surgery: OS 86.9% (HR 0.943, 95% CI 0.652-1.365; p=0.757), BCSS 94.7% - No Axillary Surgery: OS 85.2%, BCSS 96.7%   Statistical analysis by Kaplan-Meier method, log-rank test, multivariate Cox proportional hazard analysis | N/A | Axillary surgery in cN0 elderly patients does not provide a survival benefit, supporting omission | Limited diversity in population, small sample population omitting axillary surgery, lack of recurrence data, lack of detail regarding systemic therapy, comorbidity data lacking |  |
| Population based cohort | 2005-2015 | 10 years | Is axillary staging associated with survival in elderly women with breast cancer? | Women $\geq$ 70 years old with T1-2 breast cancer undergoing breast surgery in the Surveillance, Epidemiology, and End Results (SEER) Database, using propensity score weighting   - Axillary Surgery - No Axillary Surgery | Predom. ER/PR+ | *n* = 144,329 | Worse OS and BCSS in women omitting axillary staging  OS: adjusted HR 1.22, 95% CI, 1.19-1.25 compared to those who received axillary surgery  BCSS: adjusted sdHR 1.14, 95% CI 1.08-1.21 compared to those who received axillary surgery  Statistical analysis by Kaplan-Meier method, competing risk analysis | N/A | Omitting axillary surgery was associated with significantly worse OS and BCSS in elderly women with early-stage breast cancer | Lack of clinical stage, complete HER2, and comorbidity data |  |
| Population-based cohort | 2010-2016 | 6 years | What is the impact of omitting axillary surgery on elderly breast cancer patients? | Women 65-95 years old undergoing surgery for stage I/II breast cancers, identified in the Ontario Cancer Registry, survival and recurrence outcomes assessed using propensity score model   - Axillary Surgery - No Axillary Surgery | Predom. ER+, PR+, HER2- | *n* = 17,370 | No significant difference in BCSS (sdHR 0.98, 95% CI 0.77-1.25)  Worse OS (HR 1.14, 95% CI 1.04-1.25p<0.001) in patients omitting axillary surgery   - Axillary Surgery: OS 87.7% (95% CI 87.1-88.2) - No Axillary Surgery: OS 68.0% (95% CI 65.7-70.2)   Statistical analysis by weighted Cox models of OS, weighted Fine and Gray models for BCSS | N/A | Omitting axillary surgery in elderly women with early stage breast cancer is associated with worse OS, but no impact on BCSS | Incomplete tumor marker data, missing comorbidity data, missing clinical stage data |  |
